# Using a large-scale knowledge database on reactions and regulations to propose key upstream regulators of various sets of molecules participating in cell metabolism

**DOI:** 10.1186/1752-0509-8-32

**Published:** 2014-03-17

**Authors:** Pierre Blavy, Florence Gondret, Sandrine Lagarrigue, Jaap van Milgen, Anne Siegel

**Affiliations:** 1 INRA, UMR1348 PEGASE, Domaine de la Prise, 35590 Saint-Gilles, France; 2 AgroCampus-Ouest, UMR1348 PEGASE, 74 rue de Saint Brieuc, 35000 Rennes, France; 3 CNRS-Université de Rennes 1-INRIA, UMR6074 IRISA, Campus de Beaulieu, 35042 Rennes, Cedex, France

**Keywords:** Biochemical reactions, Causalities, Gene expression, Knowledge integration, Protein partners, Upstream regulators

## Abstract

**Background:**

Most of the existing methods to analyze high-throughput data are based on gene ontology principles, providing information on the main functions and biological processes. However, these methods do not indicate the regulations behind the biological pathways. A critical point in this context is the extraction of information from many possible relationships between the regulated genes, and its combination with biochemical regulations. This study aimed at developing an automatic method to propose a reasonable number of upstream regulatory candidates from lists of various regulated molecules by confronting experimental data with encyclopedic information.

**Results:**

A new formalism of regulated reactions combining biochemical transformations and regulatory effects was proposed to unify the different mechanisms contained in knowledge libraries. Based on a related causality graph, an algorithm was developed to propose a reasonable set of upstream regulators from lists of target molecules. Scores were added to candidates according to their ability to explain the greatest number of targets or only few specific ones. By testing 250 lists of target genes as inputs, each with a known solution, the success of the method to provide the expected transcription factor among 50 or 100 proposed regulatory candidates, was evaluated to 62.6% and 72.5% of the situations, respectively. An additional prioritization among candidates might be further realized by adding functional ontology information. The benefit of this strategy was proved by identifying PPAR isotypes and their partners as the upstream regulators of a list of experimentally-identified targets of *PPARA*, a pivotal transcriptional factor in lipid oxidation. The proposed candidates participated in various biological functions that further enriched the original information. The efficiency of the method in merging reactions and regulations was also illustrated by identifying gene candidates participating in glucose homeostasis from an input list of metabolites involved in cell glycolysis.

**Conclusion:**

This method proposes a reasonable number of regulatory candidates for lists of input molecules that may include transcripts of genes and metabolites. The proposed upstream regulators are the transcription factors themselves and protein complexes, so that a multi-level description of how cell metabolism is regulated is obtained.

## Background

The post-genomic era in research on biological organisms is characterized by an avalanche of data obtained on tissues or cells by high-throughput techniques like microarrays, proteomics and metabolomics [1]. These data are first statistically analyzed to produce lists of differentially-expressed molecules between experimental conditions [2,3]. A biological meaning to these differentially-expressed molecules is then searched by querying encyclopedic information on cell mechanisms and pathways [4]. For that purpose, coding genes are generally clustered based on their cellular component, molecular function, or biological process referenced in gene ontology databases; this may be automatically compiled using web tools such as DAVID [5]. A detailed analysis of these clusters can be done manually by experts reviewing a focused literature, especially when these sub-lists include well-known genes in their own domain of expertise. However, this does not quickly allow the identification of upstream regulators in the underlined pathways, nor does it provide an integrated view of the cellular responses. These aspects are difficult to attain due to the large (thousands of molecules), complex (numerous interactions) and multi-scale nature (involving genes, protein complexes and metabolites) of the system to be analyzed. The situation is also complicated by the fact that the key upstream regulator may be not included in the list of genes suggested as differentially-expressed according to a threshold probability.

Therefore, post-genomic analyses could benefit from automatic and efficient analyses of experimental data confronted to encyclopedic information. Among the existing methods, causal networks have been used to predict the propagation of regulation effects (inhibition or activation of genes) and to check consistency between variations and experimental data. These approaches allow gene network reconstructions based on microarray time-series data, mainly in a dynamic Bayesian statistical framework [6,7]; predictions of network behavior can be made based on formal approaches [8,9]. These methods are suitable for analyzing the variations between experimental conditions in which the regulators affect reaction speeds (causal dependencies). They do not take into account enzymatic reactions in which substrates are catabolized to produce other molecules. Therefore, the regulatory roles of enzymes, hormones, and protein-protein interactions in signaling cascades, are not considered.

In the present study, a method was proposed to solve this problem by modeling regulations and biochemical reactions in a new unified formalism of regulated reactions. This formalism was designed to merge and analyze any information available in various encyclopedic sources such as KEGG [10], MetaCyc [11], PathwayCommon [12] and TRANSPATH [13]. We focused on TRANSPATH database providing information on mammalian signal transduction and metabolic pathways, such as gene regulatory pathways, protein-protein interactions, and direct modifications of proteins [14]. Our method enabled the conversion of the regulated reactions in a causality graph, so that a crawling algorithm could be used to identify direct and indirect relationships between molecules. Candidates were then proposed according to their ability to regulate many targets (coverage score) or to be specific to a set of few targets (specificity score), so that an output of 50 to 100 molecules could be further considered to easily screen the upstream key regulators. The accuracy of the method was evaluated by calculating the success rate in retrieving expected transcription factors from a large number of input lists of target genes, each with a known solution. Rate of success was evaluated to 62.6% or 72.5% of the tested situations when sets of 50 or 100 regulatory candidates were considered, respectively. The method may also benefit from a post-prioritization among candidates based on the automatic addition of functional ontology information. Lastly, the ability of the method to identify upstream gene regulators from a list of metabolites participating in glycolysis was illustrated.

## Results and discussion

**Figure 1:**
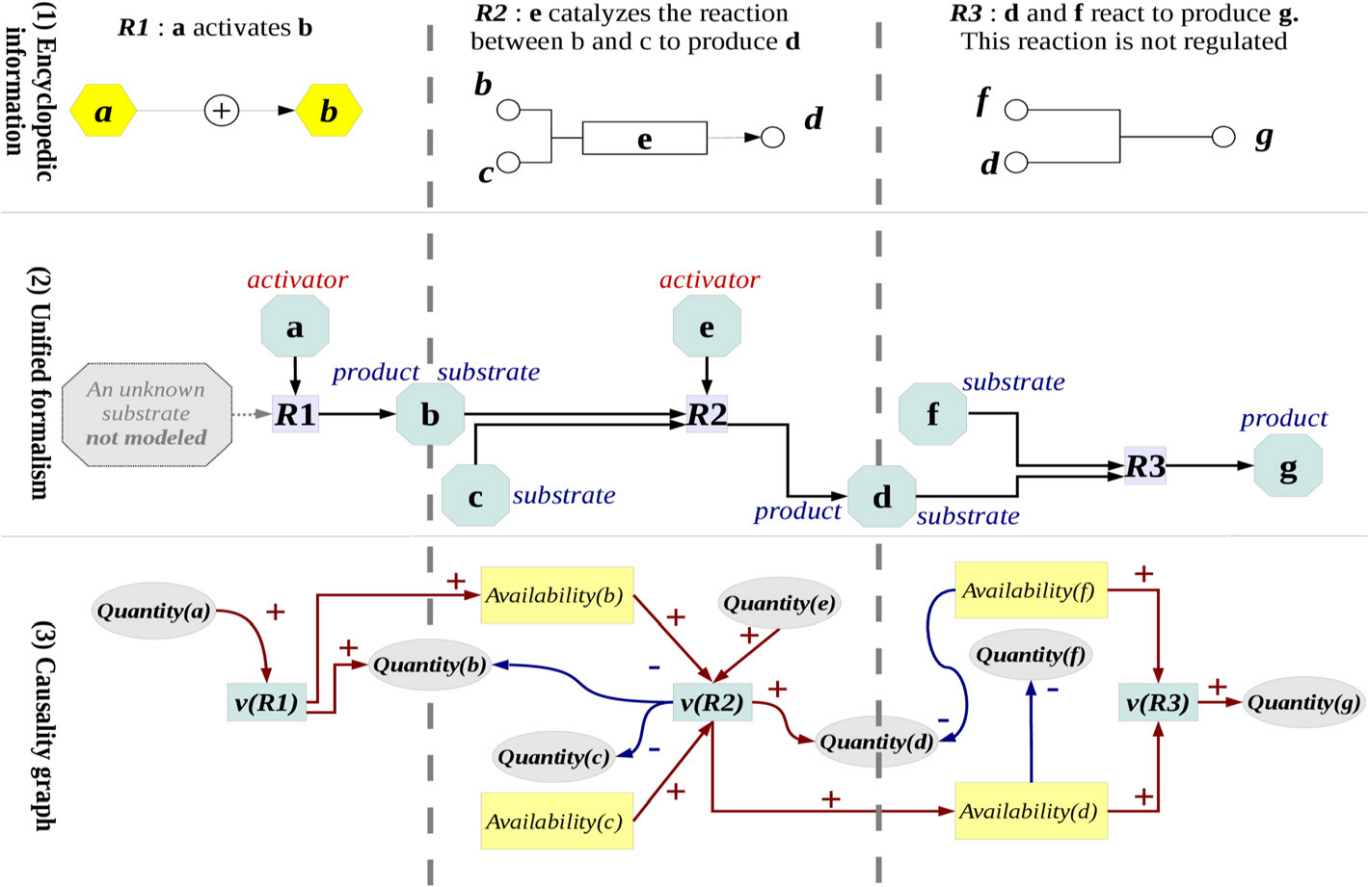
**A new unified formalism merging reactions and effects and its interpretation in a causality graph.** This graphical scheme illustrates the successive steps in the conversion of irreversible reactions available in encyclopedic database in a causality graph. (**1**) **Common knowledge:** encyclopedic information about mammalian cell metabolism and its regulation generally refer to two different concepts: i) the effects (**r1**) that describe the consequence of the variation induced by a regulator (e.g., **a**, a transcription factor; **e**, an enzyme) on reactions (e.g., rate of transcription, speed of reaction), and ii) the reactions (**r2**) that catabolize substrates (e.g., **b** or **c**) to produce newly formed molecules (products; **d**). Regulators may have a positive (activator) or a negative (inhibitor) effect, or could affect the reaction with a sign which is not yet properly referenced (modulator). (**2**) **Unified formalism:** a new common formalism of “regulated reactions” was proposed. The effects were modeled as reactions that were regulated by a regulator and that produced a regulated product using a not limiting and not modeled unknown substrate. This formalism clearly distinguished between the fluxes of substrates and products, on the one hand, and reaction speed, on the other, this latter trait being dependent on the amounts of regulators. (**3**) **Causality graph:** Under the quasi-stationary hypothesis, the reaction speed was described as an increasing function of the availability of each substrate and of the amounts of each activator, a decreasing function of the amount of each inhibitor, or a monotonous function of the amount of each modulator, respectively. The causality graph described the variations in the amounts of molecules, the speeds of reactions, and the fluxes (as nodes); it predicted their consequences as edges (positive: +, negative: - or unknown: ?). Both regulated reactions and reactions that were not explicitly regulated could be considered in this formalism.

Both regulations and biochemical reactions have been merged into regulated reactions, as illustrated in Figure 1. These regulated reactions corresponded to a set of signed relationships between substrates and products and regulators of reactions, that can be activators (positive effects), inhibitors (negative effects) or modulators (i.e., regulating a reaction with an unknown sign). Importantly, these regulators included transcription factors (TF), by assuming that the regulated gene was the product of a reaction using a non-limiting unknown substrate, and the enzymes catalyzing biochemical reactions between substrates and products. The regulated reaction set was then transformed in a causality graph to qualitatively interpret variations in the amounts of molecules, mass transfers between reactions, and reaction speeds (i.e., the nodes of the graph) in causes and effects (i.e., directed edges with positive, negative or unknown signs).

### Effects of neighborhood on graph connectivity

This formalism of regulated reactions can be theoretically applied to all encyclopedic databases; however, information was obtained from the TRANSPATH database as a case study. The causality graph obtained after converting encyclopedic information in a regulated network had a large-scale nature, with more than 400,000 nodes and 1,800,000 directed edges (Table 1). In comparison, information in TRANSPATH source included about 158,500 molecules and 224,000 reactions. This difference was related to the fact that many links were created in the causality graph when a molecule was shared by many regulated reactions, and these links were propagated throughout the graph. Edges with a positive sign outnumbered the negatives edges (Table 1), which is in agreement with the view that a large number of biochemical reactions under physiological conditions are preferentially catalyzed in one direction [15].

**Table 1:** Modeling encyclopedic information as a set of regulated reactions used to build a causality graph

**Encyclopedic information**^**a**^		**Regulated reactions**^**b**^		**Causality graph**^**c**^	
**Molecules**	**158,545**	**Nodes**	**291,306**	**Nodes**	**402,553**
Metabolites	122,591	Metabolites, genes	132,762	Quantity	92,872
Genes	35,594			Availability	151,127
		Reversible reactions	40,541	Reaction speed	158,554
		Irreversible reactions	118,013		
**Relations**	**224,080**	**Edges**	**407,966**	**Edges**	**1835,018**
Reversible reactions	41,278	Substrates	147,009	+	1711,844
Irreversible reactions	110,838	Products	168,748	-	104,538
Positive effects	2,493	Activators	72,899	?	18,636
Negative effects	854	Inhibitors	960		
Unknown effects	10,251	Modulators	18,350		
Gene to protein	59,956				

^a^Obtained from the TRANSPATH database that includes a description of biochemical reactions, protein-protein interactions, and transcription factors involved in signal transduction of mammalian cells.

^b^Reactions and effects (i.e., causal dependencies) were unified in the concept of regulated reactions, which corresponded to a set of substrates, products and regulators (activators, inhibitors or modulators). These regulators included transcription factors (assuming the regulated gene as the product of a reaction using a non-limiting unknown substrate) and enzymes (catalyzing biochemical reactions between substrates and products). A Boolean attribute was added to distinguish between reversible and irreversible reactions.

^c^The regulated reactions were converted in a causality graph to model the variations in the amounts of molecules, fluxes and reaction speeds (nodes), and to predict their consequences (edges). Because nodes were shared between various regulated reactions, the conversion of the regulated reaction network in the causality graph led to a large increase in the number of nodes and edges.

To analyze graph properties, three input lists with different number and nature of molecules (gene transcripts or metabolites) were considered. Details were provided in Additional file 1: Table S1. Irrespective of the list, the proportion of molecules and the number of reactions included in the regulated reaction networks dramatically increased with the level of neighborhood considered throughout graph computation (Table 2). This proportion was notably reduced when the original information was filtered by removing the first 100 or 1,000 molecules participating in most of the reactions. This means that the regulated reaction set was more specific to the input target list when these molecules were not taken into account during neighborhood computation. The topological properties of the network provided an explanation for this situation. Indeed, as for many metabolic networks in various organisms [15], the structural connectivity of the network (as indicated by γ values of 1.9 to 2.3; Table 2) indicates that most nodes had few links, but a few highly connected nodes (so-called “hubs” [15]) linked the rest of the less connected nodes to the system. This supports the view that this network as other large networks [16] self-organized into a scale-free structure. The network diameter (D) increased with the level of neighborhood, which was expected because new nodes were added to expand the network. Topological attributes were kept with the conversion of the regulated reaction set into the causality graph.

**Table 2:** Topological properties of regulated reaction networks and causality graphs built from various experimental lists

**Hub**^**c**^	**Dataset**^**a**^	**List 1**			**List 2**			**List 3**		
	**Neighborhood**^**b**^	**1**	**2**	**3**	**1**	**2**	**3**	**1**	**2**	**3**
0	**Regulated reactions**^**d**^									
	Molecules, %	1.9	17	52	0.8	10	46	0.1	1.8	14
	Reactions, %	1.1	16	59	0.6	9.2	53	0.05	1.6	15
	*γ*^*e*^	2.1	2.2	2.3	2.3	2.2	2.3	1.9	2.3	2.2
	*r*	84	93	96	89	90	96	92	94	94
	*L*	3.6	4.2	-	4.4	4.1	-	2.9	48	-
	*D*	6	9	-	8	14	-	4	11	-
	**Causality graph**									
	Nodes, %	1.8	19	61	0.6	10	55	0.09	1.4	16
	Edges, %	2.6	28	13	0.1	16	72	0.06	0.3	19
	*γ*	1.69	1.87	1.94	2.35	1.87	1.93	1.67	2.35	1.91
	*r*	79	78	85	91	66	85	83	94	75
100	**Regulated reactions**^**d**^									
	Molecules, %	1.4	11	44	0.8	6.9	35	0.1	1.8	12
	Reactions, %	0.8	8	49	0.6	5.7	38	0.05	1.6	11
	*γ*	2.10	2.25	2.30	2.34	2.21	2.30	1.89	2.30	1.24
	*r*	87	97	98	89	96	98	92	94	97
	*L*	3.8	4.5	7.7	4.4	4.4	-	2.9	4.8	4.4
	*D*	6	9	12	8	14	-	4	11	16
	**Causality graph**									
	Nodes, %	1.2	10	50	0.6	6.7	40	0.09	1.4	13
	Edges, %	1.3	7.1	41	0.1	3.5	29	0.06	0.3	6.6
	*γ*	1.73	1.97	2.03	2.35	2.06	2.04	2.35	2.09	1.67
	*r*	84	93	91	91	89	91	83	94	93
1000	**Regulated reactions**^**d**^									
	Molecules, %	0.7	3.8	20	0.8	3.6	16	0.1	1.8	7.1
	Reactions, %	0.4	1.9	22	0.6	2.4	14	0.05	1.6	5.8
	*γ*	2.15	2.21	2.42	2.34	2.24	2.38	1.89	2.30	2.28
	*r*	94	97	96	89	97	96	92	94	97
	*L*	3.8	4.4	-	4.4	5.0	5.1	2.9	4.8	-
	*D*	6	9	-	8	15	13	11	-	4
	**Causality graph**									
	Nodes, %	0.6	3.0	23	0.6	3.0	15	1.4	6.6	0.09
	Edges, %	0.6	2.2	8.9	0.1	1.0	5.3	0.3	2.3	0.06
	*γ*	1.72	1.88	2.34	2.35	2.28	2.03	2.35	2.35	1.67
	*r*	86	87	97	91	97	91	94	97	83

^a^Three case-study situations were analyzed by using lists with different number and nature of targets: i) the list 1 included 250 unique genes targeted by *PPARA*[20], ii) the list 2 included 136 gene transcripts that were either up- or down-regulated after addition of agonists of PPARA in cell culture [21], and iii) the list 3 consisted in seven metabolites involved in the successive steps of glycolysis in mammalian cells [32]. Details are provided in Additional file 1: Table S1.

^b^The first level neighborhood was obtained by taking all the reactions in which the input molecules were involved. The second level was obtained by adding all the new molecules involved in these latter reactions. The third level was an iteration of this procedure.

^c^Throughout the neighborhood computation, the first *n* molecules involved in most reactions in cell metabolism were ignored. In the tested situations, *n* corresponded to 0, 100 or 1,000. After neighborhood computation, these molecules (so-called “hubs” because they shared many relationships) were added to the network only in the case where they participated in reactions selected from the input lists.

^d^The proportion (%) of molecules and reactions (nodes and edges, respectively) that were selected from the full graph in the regulated reaction network (causality graph, respectively) was calculated.

^e^The topological properties of the network were analyzed using different network statistical parameters. A *r* value close to 1 indicates that the graph was scale-free. Assuming that the probability *P*(*k*) that a molecule in a network interacts with *k* other molecules follows a power law [*P*(*k*) ~ *k*^*-γ*^], a high *γ* value indicates that there were few highly connected nodes in the network. The quantity *L* denotes the average shortest path length by which one can reach node *A* by node *B*, and *D* corresponds to the graph diameter. Within a row, the sign “–“ indicates that the computation of these parameters had failed. This analysis shows that the conversion of regulated reactions in a causality graph kept the original structure of the network.

### Accuracy of the method to provide the expected transcription factor among a reasonable set of regulatory candidates

To address the accuracy of the method in proposing relevant upstream regulatory candidates, it is important to consider different lists of target genes, each having a known solution. For that purpose, 250 different lists of target genes (Additional file 2: Table S2) that are referenced in the Transcriptional Regulatory Element Database (TRED, [17]) as being controlled by a given TF in human, mouse or rat, were submitted. Success was attributed only to tests in which the known solution was present among a reasonable set of candidates having the highest scores for coverage or for specificity, respectively. As expected, the rate of success increased with the number of candidates considered in the output lists (Table 3) to reach a maximum of 87.7% in situations where sets of 1,000 molecule candidates were retained. Importantly, the known TF was included in the output lists of candidates whether or not the TF was present or absent in the input lists of targets. Together, this provides evidence that the formalism of regulated reactions transposed into causality graph is suitable for the identification of upstream key regulators. Success was better when the specificity score was considered. It was also improved when not only “gene” terminology but also “molecule” terminology (including proteins, complexes, etc.) in the TRANSPATH database were considered for solutions (Table 3). Finally, the rate of success was still reasonable when small sets of specific molecule candidates (50 to 100) were considered (62.6% and 72.5% of the tested situations, respectively). This means that this method was able to curate a reasonable number of potential regulators among the ~1,850 TF and the ~20,000-25,000 protein coding genes in the human genome [18]. The rate of success fell to 6.8% and 10%, respectively, when lists were randomly swapped to calculate a sub-graph of causalities from biologically irrelevant lists of targets. This demonstrates that few regulatory candidates could be identified by chance only. In some tests considered as failed, the set of retained candidates included isotypes rather than the expected TF (e.g., *RARB* and *RARG* instead of *RARA*). This is likely because many targets were shared by the various isotypes (e.g., 77.5% and 90.5% of target genes regulated by *RARB* and *RARG*, respectively, were included in the list of *RARA*-regulated genes; [17]). In the majority of the failed tests, many molecules shared similar scores in the answer set. This means that users had to consider a trade-off between a reasonable rate of success and the time needed to review a dedicated literature to prioritize among a large number of candidates. The number of candidates in the answer set can be chosen at each computation, but we recommend retaining sets of 50 to 100 candidates as good trade-offs.

**Table 3:** **Success in retrieving the known transcription factor regulating an input list of its gene targets**^**a**^

**Lists of target genes**^**b**^				
**Number of regulatory candidates considered**^**c**^	**Number of tests (%) where the known TF was found among proposed gene candidates**		**Number of tests (%) where the known TF was found among proposed molecule candidates**	
	**According to coverage score**	**According to specificity score**	**According to coverage score**	**According to specificity score**
1	11.9	12.2	13.2	14.4
10	36.3	35.9	39.0	40.3
20	44.1	45.1	47.8	49.9
**50**	**54.9**	**57.7**	**59.1**	**62.6**
**100**	**66.7**	**67.1**	**70.7**	**72.5**
200	75.5	75.9	78.8	79.7
500	82.5	82.6	86.2	86.5
1000	83.7	83.7	87.6	87.7
**Lists of randomly-shuffled genes**^**b**^				
**Number of regulatory candidates considered**	**Number of tests (%) where the known TF was found among proposed gene candidates**		**Number of tests (%) where the known TF was found among proposed molecule candidates**	
	**According to coverage score**	**According to specificity score**	**According to coverage score**	**According to specificity score**
1	0.0	0.0	0.0	0.4
10	0.4	1.2	1.6	2.4
20	0.4	1.2	2.4	4.0
**50**	**2.4**	**3.2**	**6.8**	**8.0**
**100**	**4.0**	**5.6**	**10.0**	**13.2**
200	5.6	7.2	14.8	18.8
500	11.6	13.2	27.2	28.0
1000	20.0	21.2	38.4	38.8

^a^The rate of success corresponds to the number of situations out of 250 tests, where the known transcription factor (TF) referenced in the transcriptional regulatory database (TRED) [17] was present among n regulatory candidates that were automatically provided. Candidates were scored for coverage (i.e., the ability to explain the greatest number of targets) or for specificity (i.e., a tradeoff between the number of regulated targets and the total number of regulated molecules). Because the known TF can have the same score as a set of other candidates (i.e., *ex aequo*), the probability to find the known TF among the candidates was estimated under the hypothesis that *ex aequo* candidates were randomly ordered. The results indicate that rate of success increased with the number (n) of candidates considered.

^b^A total of 250 different lists of genes, each of these lists being regulated by a known transcription factor, were automatically extracted from TRED. These lists contained between 1 to 352 target genes (see Additional file 2: Table S2). In a first step, each list of genes targeted by a known TF was successively submitted to analysis. In a second step, all genes included in these lists were randomly shuffled to constitute 250 lists of biologically non-relevant targets.

^c^The results indicate that the rate of success was reasonable when 50 to 100 candidates in the answer sets were considered (as indicated in bold face).

### Proposed candidates other than the expected TF might be relevant upstream regulators of experimentally-derived lists of target genes

Because the overlap between experimentally-obtained target genes and the targets reported in the majority of knowledge libraries is surprisingly small [19], proposing candidates other than the expected TF may be biologically relevant. To further examine this aspect, two lists of target genes (see Additional file 2: Table S2) that have been experimentally-demonstrated [20,21] to respond to *PPARA*, a TF that regulates various aspects of fatty acid metabolism and storage, were analyzed. Irrespective of the score used, it is first important to note that *PPARA* was successfully proposed among an examined answer set of 50 regulatory candidates (Table 4), with one noticeable exception where *PPARA* was included in a non-dissociable set of 2,193 candidates sharing a similar score. Among the candidates, isotypes delta (*PPARD)* and gamma (*PPARG)* were elicited with scores similar to those assigned to *PPARA*. They could be considered as true positive candidates, because microarray analysis of cell lines ectopically expressing PPAR family members [22] had shown that many of the established PPAR target genes are equally receptive to all 3 receptors although preferential targets do exist for each isotype (27 for *PPARG*, 33 for *PPARD* and 93 for *PPAR*A, respectively, out of a total of 284 identified targets). The same situation applies to retinoid X receptors (*RXR*), because RXRs are well-known obligate heterodimer partners for PPAR actions in controlling the storage and use of energy, and they play integrative roles across multiple metabolic systems [23]. Less expected, vitamin D receptor (*VDR*) and the heterodimer complex VDR-RXRA were also found (Table 4). In support of this finding, a cross-talk between VDR- and PPAR-signaling pathways in modulating gene expression has been reported in various cell types and VDR and PPARs compete for a predominant hetero-dimerization with their RXR partners [24]. Many of the proposed candidates having the highest scores for coverage or for specificity could also be biologically relevant, because they participated in pathways closely related to *PPARA* actions on lipid metabolism [25]. This concerns lipid metabolism itself (*SREBP1A*, *GPD1*, *LEPR*, *PTE1, ACAD8, CES3*), cholesterol homeostasis (*SREBP2, NPC1, SLC10A2*), fatty acid transport and lipid transfer (*SLC27A1*, *PCTP, LRP4*) and peroxisomal oxidation (*HDS1B4*, *HACL1*). Finding candidates involved in the elongation and desaturation of fatty acids (*FADS1*, *ELOVL5* and *ELOVL6)* was also biologically relevant, because long-chain polyunsaturated fatty acids are *PPARA* agonists [26]. Lastly, the presence of the macrophage antigen *CD68* in the answer set matches with the observation that adipose tissues of PPAR alpha-null mice exhibited an up-regulation of *CD68* mRNA [27]. Importantly, none of these candidates were controlled themselves by PPARA [17].

**Table 4:** **PPARs and other regulatory partners were automatically proposed from experimental lists of *PPARA *** **gene targets**

**Input list 1**^**a**^		**Input list 2**^**a**^		**Input list 1**^**a**^		**Input list 2**^**a**^	
**Score: coverage**^**b**^				**Score: specificity**^**b**^			
**RXR**				**RXR**			
RXRA: PPARG	[4-29]^c^	RXRA	[2-20]	RXRA: PXR-isoform1A	[1]	RXRA: PPARA	[4]
RXRA: PPARA	[4-29]	RXRA: VDR	[2-20]	RXR: VDR	[3]	RXRA: PPARD	[5-8]
RXRA: PPARD	[4-29]			RXRA: PPARD	[4]	RXRG: PPARA	[5-8]
RXRG: PPARA	[4-29]			RXRA: PPARG	[14]	RXRG: PPARD	[5-8]
RXRG: PPARD	[4-29]					RXRA: PPARG	[9,10]
RXRG: PPARG	[4-29]					RXRG: PPARG	[9,10]
RXRG: PPARG	[4-29]						
**VDR**				**VDR**			
VDR	[30-2223]	VDR	[2-20]	VDR: RXRA	[3]		
VDR: RXRA	[30-2223]	VDR: RXRA	[2-20]				
VDR: calcitriol	[30-2223]						
VDR: calcitriol: 9-cis-retinoic acid: RXRA	[30-2223]						
VDR: BLM	[30-2223]						
**PPARA**				**PPARA**			
PPARA: RXRA	[4-29]	PPARA	[21-1702]	PPARA: RXRA	[2]	PPARA: RXRA	[4]
PPARA: RXRG	[4-29]	PPARA: RXRA	[21-1702]				
**PPARG**				**PPARG**			
PPARG: RXRA	[4-29]	PPARG: abietic acid	[21-1702]	PPARG: RXRA	[14]	PPARG: RXRA	[9,10]
PPARG: RXRG	[4-29]	PPARG: 15d-PGJ2	[21-1702]			PPARG: RXRG	[9,10]
		PPARG: azPC	[21-1702]			PPARG	[12]
**PPARD**				**PPARD**			
PPARD: RXRA	[4-29]	PPARD	[21-1702]	PPARD: RXRA	[4]	PPARD: RXR	[5-8]

^a^Detailed lists are provided in Additional file 1: Table S1, with i) list 1 including 250 regulated genes identified as controlled by *PPARA* from a literature review [20] and ii) list 2 including 136 differentially expressed genes in response to *PPARA* agonists in cell culture [21].

^b^Regulatory candidates of these lists were elicited by an automatic algorithm based on encyclopedic information extracted from the TRANSPATH database and modeled as a causality graph. The candidates were scored for coverage (i.e., the number of targets regulated by a given candidate) or for specificity (i.e., a tradeoff between the number of regulated targets and the total number of regulated molecules) and the first 50^th^ candidates having the highest scores were retained.

^c^The range [*N*_*i*_-*N*_*j*_] indicated that the genes or the protein complex in which they participated had the same score as a set of *N*_*j*_*- N*_*i*_*+1* molecules. When a molecule or a complex appeared more than once, only its best position was shown.

### Prioritizing upstream regulators could be done by functional clustering

Because the proposed candidates may be included within a non-dissociable set of molecules having same scores, an ultimate step in our method was to evaluate the benefit of automatically adding functional information to candidates. For that, a dedicated web-service available online without fees [28] can be used. The results show that the input list of regulated transcripts and the output list of proposed regulatory candidates shared clusters related to fatty acid and cholesterol metabolisms (Table 5), which was expected considering roles of PPARs [22,29]. Importantly, clusters included fewer genes when calculated from the list of candidates than from the input list of regulated genes. At least one PPAR isotype was apparent in clusters calculated from the candidate list, which was not the case when clusters were calculated from the regulated transcripts. Moreover, a new cluster termed PPAR signaling pathway including the three PPAR isotypes and their heterodimer partners RXRs, was apparent in the answer list (Table 5). The method had also found candidates for clusters related to cell adhesion, migration, and developmental process. Among these, *TGFB1* is considered to regulate numerous cell adhesion processes including cell proliferation, differentiation, motility, and apoptosis [30]. This gene is also a known target of oxysterols and various lipid compounds [31], which may give an explanation for its identification as a regulatory candidate from the list of *PPARA*-responsive genes. Altogether, these results indicate that our method coupled with automatic functional annotation is able to select a small subset of genes regulating various functions originally represented among the regulated genes. It provides the biologist with a clear synthesis of the information and relevant upstream candidates across the clusters.

**Table 5:** Functional clusters among target genes or their regulatory candidates

**Regulated targets**^**a**^	**Regulatory candidates**^**b**^
**Fatty acid metabolism**^**c**^	
Cluster 1: *ACAA2, ACADL, ACADVL, ACSS2, ALDH9A1, CLU, DECR1, DGAT2, DHRS4, ECH1, ARCC1, FNTB, HADHB, HMGCS2, LSS, MBLN3, NME4, PAPSS2, PDK1, PEX11A, PNPLA2, PYCR1, RETSAT, UCP2*	Cluster 1: *PNPLA2,****PPARA, PPARG,****SERPINA3, TGFB1*
Cluster 2: *ACADL, ACADVL, ALDH9A1, DECR1, DHRS4, EGLN3, PYCR1, RETSAT*	Cluster 2: *ACAA2, ACADL, ACADVL, ECH1, HADHB, PNPLA2,****PPARA, PPARD***
**Cholesterol metabolism**	
Cluster 4: *ACAA2, ACSS2, DGAT2, HMGCS2*	Cluster 3: *ACAA2, ACSS2, DGAT2, HMGCS2, LSS,****PPARD, ****RXRA*
**Cell adhesion and migration**	
Cluster 7: *CDH11, CLU, COL18A1, DPT, LAMA2, LAMA4, PTK7, SERPINE2*	Cluster 4: *COL18A1, LAMA2, LAMA4, SERPINE2, TGFB1*
**Cell development**	
Cluster 3: *SERPINA3, SERPINE1, SERPINE2*	Cluster 6: *CLU, COL18A1, DPT, EGLN3, FNTB,****PPARD, PPARG, RXRA****, SERPINE1, TGFB1*
	Cluster 5: *EGLN3, LAMA2, LAMA4,****PPARD, PPARG, RXRA, RXRG, ****TGFB1*
	Cluster 8: *CLU, JUNB, JUND****, PPARD, PPARG, ****TGFB1*
**Cell signaling**	
Cluster 6: *APCDD1, CA6, CDH11, CLU, COL18A1, DGAT2, DPT, HTRA3, LAMA2, LAMA4, LCN2, LGALS9, NPR3, PNPLA2, OPDC3, PTK7, RETSAT, SERPINA3, SERPINE1, SERPINE2, STRA6*	Cluster 7: **(PPAR signaling pathway)** : *ARNTL, ARNTL2, CLOCK, CLU, COL18A1, DPT, GLN3, ERCC1, FNTB, HLTF, HMGCS2, JUNB, JUND, LAMA2, LAMA4, MBNL3, PAPSS2, PNPLA2,****PPARA, PPARD, PPARG, RXRA, RXRG,****SERPINA3, SERPINE1, TGFB1*
**Peroxisome microbody**	
Cluster 5: *DHRS4, ECH1, PEX11A*	
Cluster 9: *CDH11, JUND, NPR3, PAPSS2, TGFB1*	

^a^Transcripts experimentally identified as responsive to *PPARA* agonists in cell culture [21] (see Additional file 2: Table S2 for details) were clustered using the DAVID functional annotation tool [5]. Only clusters with an enrichment > 0.5 and a Benjamini score < 0.15 were kept. Eventually, a synthetic description was chosen to name the cluster.

^b^Fifty upstream regulatory candidates that were automatically-proposed according to best specificity scores (see Additional file 2: Table S2 for a detailed list) were clustered. All molecules were first linked to their related genes, with heterodimer protein complexes (e.g., PPARA:RXRA) being switched in the two genes (e.g., *PPARA* and *RXRA*).

^c^The results show that the input list of regulated transcripts and the output list of proposed candidates notably shared clusters related to fatty acid and cholesterol metabolisms. All clusters were enriched in transcription factors, and especially in peroxisome proliferator-activated receptors (PPAR) isotypes and their partners RXR (indicated in bold face) when calculated from the list of regulatory candidates.

### Upstream integrative candidates were proposed from a list of up-regulated metabolites

**Figure 2:**
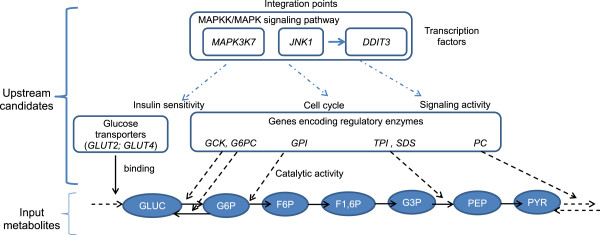
**Finding upstream regulators of various metabolites.** This scheme illustrates how an input list of metabolites and the output list of regulatory candidates at the enzymatic and molecular levels were positioned together. A list of 7 metabolites participating in glycolysis (GLUC: glucose; G6P: glucose-6-phosphate; F6P: fructose-6-phosphate; F-1,6P: fructose 1,6 diphosphate; G3P: glyceraldehyde-3 phosphate; PEP: phosphoenolpyruvate; PYR: puryvate) were submitted as input (i.e., molecules for which common or specific upstream regulators should be identified). All these metabolites were supposed to be increasingly abundant in response to an unknown external factor. Regulated reactions in which these metabolites were involved and their neighbor molecules (up to 3 levels of neighboring) were extracted from an encyclopedic database on mammalian signaling pathways and converted into a causality graph. The proposed candidates included transporters (glucose binding; GLUT2/*SLC2A2* and GLUT4/*SLC2A4*), catalytic enzymes (*GCK*: glucokinase; *GPI*: glucose-6 phopshate isomerase; *TPI*: triose phosphate isomerase; *PC*: pyruvate carboxylase; *G6PC*: glucose-6 phosphate carboxylase; *SDS*: serine dehydratase) and integrative actors (*MAP3K7*; *MAPK8/JNK1*) in glucose homeostasis and insulin signaling.

In the present study, the formalism had merged biochemical reactions with gene regulations. Therefore, it is meaningful to illustrate the possibility offered in analyzing a list of metabolites to retrieve molecular regulators. Seven metabolites involved in the successive steps of glycolysis [32] were submitted as regulated targets. A list of 21 unique gene candidates was proposed to explain an increased abundance in these metabolites (Table 6). They included genes encoding two facilitated glucose transporters GLUT (*SLC2A2* and *SLC2A4*), which are well-known regulators of glucose uptake in eukaryotic cells [33]. Genes coding for 9 enzymes that catalyze the progression of glycolysis and neoglucogenesis (the reverse pathway of glycolysis) or were associated with other minor glucose pathways, were also included in the answer set. In addition, *MAP3K7, MAPK8/JNK1* and *DDIT3* are recognized as playing important roles in upstream regulation of glucose metabolism, because they participate in the MAPKK/MAPK pathway acting as an integration point for multiple biochemical signals. Especially, *MAP3K7* (*TAK1*) controls a variety of cell functions including transcriptional regulation; it stimulates *MAPK8/JNK1*[34], which itself plays a crucial role in controlling systemic glucose and lipid metabolism [35]. Expression of the transcription factor *DDIT3* after MAPK signaling is also able to regulate glucose homeostasis [36]. Thus, merging biochemical reactions with gene regulations allows an integrated view of regulators acting in glucose homeostasis (Figure 2). Other proposed candidates might also be biologically relevant, when considering glucose starvation as a cellular stress activating the heat-shock proteins (*HSPA5*) and other minor glucose pathways such as glucosamine synthesis (*GNPDA1*; [37]).

**Table 6:** **Upstream candidates of a list of metabolites participating in glycolysis**^**a**^

**Upstream regulatory candidates**^**b**^		
**Official symbol**	**Full name**	**Main GO process**
		**Enzymes**
*TPI*	Triose-phosphate isomerase 1	Carbohydrate metabolic process
*PC*	Pyruvate carboxylase	Carbohydrate metabolic process
*GCK*	Glucokinase (hexokinase)	Carbohydrate metabolic process
*G6PC*	Glucose-6-phosphatase	Carbohydrate metabolic process
*GPI*	Glucose-6 phosphate isomerase	Carbohydrate metabolic process
*AKR1B1*	Aldose reductase	Carbohydrate metabolic process
*SDS*	Serine dehydratase	Gluconeogenesis
*GBA*	Glucosidase	Carbohydrate metabolic process
*GNPDA1*	Glucosamine-6-phosphate deaminase	Carbohydrate metabolic process
*AK1*	Adenylate kinase	ATP metabolic process
*CAT*	Catalase	Cellular response to growth factor stimulus
		**Protein binding**
*SLC2A2*	Facilitated glucose transporter member 2 (GLUT2)	Carbohydrate metabolic process
*SLC2A4*	Facilitated glucose transporter member 4 (GLUT4)	Carbohydrate metabolic process
*HSPA5*	heat shock 70 kDa protein 5 (glucose-regulated protein)	activation of signaling protein activity involved in unfolded protein response
*GRIN1*	Glutamate receptor	Calcium ion transport
*WSF1*	Wolfram syndrome 1	Calcium ion homeostasis, glucose homeostasis
*BCL2*	B-cell CLL/lymphoma 2	B-cell homeostasis
		**Integration points**
*MAPK8*	Mitogen-activated protein kinase 8 (JNK1)	JNK cascade
*MAP3K7*	Mitogen-activated protein kinase kinase kinase 7	Activation of MAPK activity; JNK cascade
		**Transcription factor binding**
*DDIT3*	DNA damage inducible transcript 3	Negative regulation of transcription, DNA-dependent; activation of signaling protein activity involved in unfolded protein response
*XBP1*	X-box binding protein	Activation of signaling protein activity involved in unfolded protein response

^a^The input target list included the 7 metabolites in the successive steps of the glycolytic pathway in mammalian cells [32]: Glucose; Glucose-6-phosphate; D-fructose-6-phosphate; Fructose-1,6-biphosphate-1; D-glyceraldehyde-3-phosphate; Phosphoenol-pyruvate and Pyruvate. They were supposed to have an increased abundance (i.e., a positive sign) in the situation tested.

^b^The regulatory candidates were proposed from the confrontation of the target list to encyclopedic information within a causality graph. Gene ontology (GO) terms for biological process and function were then associated.

## Conclusions

A new formalism has been proposed to model encyclopedic information on molecular and cellular mechanisms into regulated reactions. A causality graph associated with a scoring procedure was used to propose upstream regulators from various sets of differentially-expressed targets that may include gene transcripts as well as metabolites. This method was also compatible with the classical causality networks [9]. Therefore, it can be used for sign prediction in further works.

## Methods

### The unified formalism of regulated reactions was proposed to integrate biochemical reactions and regulatory effects

Both biochemical reactions and gene expression regulations coexist in databases collecting encyclopedic information on regulatory networks. The reactions refer to the production or catabolism of one or more molecules. The regulations describe the effects (positive or negative dependencies or unknown sign) between the variations in the amount of one molecule on the production rate of another. To merge these two objects, a new formalism of regulated reactions was proposed. Each molecule was first assigned to be either a substrate (a molecule that is catabolized), a product (a molecule that results from the catabolism of substrates), an activator (a molecule allowing the reaction to proceed faster, like an enzyme or a transcription regulator), an inhibitor (a molecule having a negative effect on reaction speed) or a modulator (a molecule controlling reaction speed but with an unknown sign). A Boolean attribute was included to indicate whether the reaction was reversible or irreversible. In this formalism, a regulatory effect from a molecule source *a* to a molecule target *b* was thus modeled as an irreversible reaction producing *b* from the catabolism of an unknown non-limiting substrate (not modeled) and regulated by *a*. This formalism allowed a clear distinction between mass transfers within reactions (from substrates to products) and regulators of the reactions (activators, inhibitors or modulators), which were considered to be not catabolized in these reactions. Rules for modeling reactions and causal interactions into the regulated reactions are illustrated in Figure 1.

Although this formalism is suitable to model information from various libraries, the present study was based on the TRANSPATH database [13] (v2009.2). Because different species (*Homo sapiens*, *Rattus norvegicus* and *Mus musculus*) are included in this database, species-specific reactions having the same sets of substrates and products were merged into a single reaction, considering that all the regulators of the original reactions regulated the new unified generic reaction. When at least one of the species-specific reactions was indicated as reversible, the unified generic reaction was then considered as reversible. Some molecules could be considered as not limiting and highly generic in many reactions, so they were removed from the reaction sets: ATP, ADP, NTP, NDP, protein remnants, phosphate, Coenzyme A, water and H +.

### A causality graph to interpret the regulated reactions

The regulated reactions summarized the encyclopedic information about cellular mechanisms, but they did not distinguish between the causes behind and the consequences of variations in the amounts of molecules. Therefore, a set of rules was introduced to model the regulated reactions in a causality graph. More precisely, elasticity coefficients [38,39] representing the partial derivative of the reaction speed to the amounts of molecules involved in the reaction, were qualitatively studied. In the causality graph, they were introduced as signed edges to model the consequences of the variations in nodes. This allowed propagating the local effects of reactions and regulations without considering any restriction associated with their global effects on the system dynamics. Therefore, this model might be considered as an over-parameterization of all possible behaviors of the system. In this approach, all reactions were considered as irreversible. This strategy was supported by the fact that most reactions, originally described as reversible, represent the formation of a protein complex (i.e., A + B - > AB) which is not produced anywhere. Thus, the product steady-state was appropriately modeled in the causality graph, because it was directly connected to the steady-state of its substrates.

To describe the reaction speed of each regulated reaction *R*, a node *v(R)* was introduced in the causality graph. For each molecule *M*, a node *quantity(M)* was introduced to model the amount in the molecule; it was assumed to be constant at the quasi-stationary state of the system, but might vary when the system was perturbed. The main feature of the forthcoming method consisted in propagating external perturbations throughout the system to identify signs of variations in the node *quantity(M)*. For that purpose, it was necessary to take into account the rate of production of *M.* A node *availability(M)* was introduced in the graph for each molecule *M* which was a substrate of a least one regulated reaction. This node was the sum of all reaction speeds producing *M*. For each regulated reaction *R*, *v(R)* was assumed as an increasing function of the availability of all substrates and of the quantity of activators in this reaction, a decreasing function of the quantity of inhibitors, and a monotonic function of the quantity of modulators. Therefore, a positive influence was built from the node *availability(M)* to the node *v(R)* for each substrate *M*. A positive influence (respectively, negative or unknown influence) was added between the node *quantity(M)* and the node *v(R)* for each activator *M* (respectively, inhibitor or modulator). For each product *M*, the effect of an increase in the speed of the reaction *R* on the production rate of *M* was modeled by adding a positive influence between the node *v(R)* and both the node *quantity(M)* and the node *availability(M)* within the reaction.

In the case where a reaction *R* was explicitly controlled, the effect of increasing speed of the reaction on the steady-states of substrates was modeled by adding a negative influence from the node *v(R)* to the node *quantity(M)* of the substrates. In the other case, the reaction speed was supposed to be limited by the availability of at least one of the substrates participating in the regulated reaction. Therefore, for each couple of substrates (*M1*, *M2*), a negative influence was built from the node *availability(M1)* to the node *quantity(M2),* as illustrated in Figure 1. Altogether, this formalism allowed a clear distinction between the quantity of a molecule *M* and the rate of production of this molecule *M*. Therefore, the graph of influences described two main features of the system: i) the regulations upon steady-state concentrations of intermediary metabolites were modeled by both positive and negative influences over the nodes *quantity(M*), and ii), the effects of variations in the rate of production of metabolites were propagated by paths along the nodes *availability(M)*.

### Finding potential regulators from lists of molecules

A dedicated algorithm was computed to automatically provide potential upstream regulators (i.e., output candidates) from lists of regulated targets (input lists). Molecules that were able to regulate (directly or indirectly) sets or subsets of the input targets were considered as candidates. The method was based on the identification of at least one consistent path between the candidates (output) and the targets (input), *via* other molecules shared by the regulated reactions in the causality graph. First, the method selected a set of regulated reactions that were specifically related to the targets. All regulated reactions that included at least one molecule in the input list were included in the model, with the noticeable exception of molecules that were involved in a very large number of reactions. In the tested situations, the first n molecules (with n = 0; 100; or 1,000) involved in most reactions were ignored to study the topological properties of the graphs. These molecules were called “hubs” in reference to Jeong and colleagues [15]. This step was repeated two times to ensure a maximum neighboring of 3 reactions between the input list and the resulting model. The consequences of this neighbor-based pruning and hub removal on the graph topology were analyzed according to methodology described for metabolic networks [15]. Then, the resulting model of regulated reactions was transcribed into a causality graph using the rules described above.

To convert the causality graph into an explanatory graph, each node *N* in the causality graph (i.e., *quantity(M), availability(M)* or *v(R)*) was first split in two new nodes [*N,*+] and [*N,*-] in the explanatory graph. When a molecule *M* in the input list was experimentally observed as being up-regulated (respectively, down-regulated), the node [*quantity(M), -*], (respectively, [*quantity(M),+*]), was removed from the explanatory graph. Edges of the explanatory graph were exported from the causality graph to propagate effects of signs over the variations of the nodes. A node [*N*_*1*_*,+*] of the explanatory graph (respectively, [*N*_1_, -]) was said to have a positive influence over a node [*N*_*2*_*,+*] (respectively, [*N*_2_, -]) if a positive path (i.e., a path where there was an even number of negative signs) existed in the causality graph from *N*_*1*_*to N*_*2*_*.* A node [*N*_*1*_*,+*] of the explanatory graph (respectively, [*N*_1_, -]) was said to have a negative influence over a node [*N*_*2*_*,-*] (respectively, [*N*_2_, +]) if a negative path (i.e., a path where there was an odd number of negative signs) existed in the causality graph from *N*_*1*_*to N*_*2*_*.* If there was at least one path with an unknown sign, it was considered that the set of paths was of an unknown sign. Influences among the graph were computed using a crawling algorithm over the transitive closure of the causality graph.

### Scoring potential candidates explaining the variations in the amount of input molecules

Two scores were computed and assigned to each candidate. The score for coverage was defined as the number of molecules *M* for which the nodes [*quantity(M),+*] *or* [*quantity(M),-*] had an influence on the node [*quantity*(*M*_1_), +] (respectively, the node [*quantity*(*M*_1_), -]. A hyper-geometric test allowed estimating the probability *p* of sharing a greater number of targets than that obtained after a random shuffling of the set. In other words, consider an urn containing a ball for each gene in the graph, and paint white the balls representing input genes and paint black the rest of the genes. The hypergeometric distribution is a discrete probability distribution that describes the probability of *k* successes (i.e., drawing white balls) in a sequence of *n* draws without replacement from this finite population of size *N* (the number of all balls in the urn) containing exactly *K* successes (the number of white balls). Thus, a candidate regulating many elements but few targets was associated with a high *p* value in the hypergeometric test. The score for specificity was defined as the product of the score for coverage with the probability *(1-p)*. These scores were first computed both for an increase or a decrease in the quantity of *M*_*1*_. Then, if the molecule *M*_*1*_ was observed as being up-regulated (respectively, down-regulated) between experimental conditions, the final coverage score for the explanations of *M*_*1*_ was defined as the coverage score associated with an increase of *M*_*1*_ (respectively, a decrease of *M*_*1*_). For molecules *M*_*1*_ without any information on their sign of variation, the final coverage score for the explanations of *M*_*1*_ was defined as the highest score between coverage scores associated with the increase or the decrease in the quantity of *M*_*1*_. The same procedures were applied for the specificity score.

### Accuracy of the method in providing a known solution within a set of candidates

To address the accuracy of the method in retrieving upstream regulatory candidates, it is important to use different lists of targets having a known solution. For that purpose, a script and query interface using webpages were first developed to extract all lists of regulated genes and their key transcription factor (TF) in the transcriptional regulatory element database (TRED), a resource for gene regulation and functional studies in human, rat and mouse [17]. A test situation consisted in submitting a list of target genes and extracting the corresponding set of retained regulatory candidates. The test was considered successful if the TF referenced in TRED as regulating this list of targets was retrieved among *n* candidates having the highest scores for coverage or for specificity. When the expected TF was absent from this list, the test was considered as a failure. Because the searched TF can have the same score as a set of other candidates (i.e., *ex aequo*), the probability to find the known TF among the candidates was estimated under the hypothesis that *ex aequo* candidates were randomly ordered. This probability was equal to the number of *ex aequo* in the retained list divided by the total number of *ex aequo*. Although a total of 262 lists were available in TRED, 12 out of these lists corresponded to TF which could not be mapped in the TRANSPATH database. Therefore, the rate of success of the method in providing the known solution was first calculated using a total of 250 different lists of gene targets. Second, the score of success was also calculated when all regulated targets in these lists were randomly shuffled between lists in order to constitute lists of biologically non-relevant targets. In all situations, hubs (i.e., the first 1,000 molecules involved in most reactions) were temporarily ignored in the construction of the reaction network. A level of neighboring of 3 was retained for the analysis of the causality graph.

### Evaluation of solutions when experimentally-derived lists of targets were used

Experimental lists including many transcripts which have been demonstrated as responsive to *PPARA*, a TF involved in fatty acid metabolism, were used as case-studies (Additional file 2: Table S2). The first list consisted of 250 genes identified by a literature review as targets of PPAR in liver of human and mice [20]; half of these genes have been annotated as being up or down-regulated. The second list consisted in 136 gene transcripts that were proved to be responsive to *PPARA* agonists in NIH3 cells [21]. Sets of candidates scored according to coverage or specificity were then examined for the presence of the expected *PPARA* and the biological relevance of other candidates. For that purpose, dedicated literature was manually reviewed for most of these candidates. In a second step of analysis, functional annotation was added to candidates to obliterate the problems of graph connectivity in retrieving a reasonable set of candidates in some situations. Categorization of a set of 50 proposed regulatory candidates based on specificity score (see Additional file 3: Table S3) was performed using the web-accessible DAVID functional annotation tool [5]. All molecules were manually linked to their related genes, whereas heterodimer protein complexes in the list of candidates (e.g., PPARA:RXRA) were switched into two genes encoding the corresponding partners (e.g., *PPARA* and *RXRA*). Only the clustered terms with an enrichment > 0.5 and a Benjamini score < 0.15 were kept, and clusters were named by a synthetic description. The same procedure was then applied to the input list of the 136 gene transcripts identified as differentially-expressed in response to PPAR agonists [21]. The clusters from answer set of candidates and from input list of target genes were then compared.

### What about lists of metabolites?

To illustrate the possibility offered by the method to unravel lists of molecules other than genes or their transcripts, a short list of metabolites representing key steps in glycolysis was submitted to analysis. These metabolites were referenced in the KEGG database [32]: glucose itself, glucose-6-phosphate (G6P), beta-D-fructose-6-phosphate (F6P), fructose-1,6-biphosphate-1 (F1,6P), D-glyceraldehyde-3-phosphate (G3P), phosphoenolpyruvate (PEP) and pyruvate (PYR). All these metabolites were supposed to have an increased abundance in response to an unknown external factor, so that a positive sign was added to each metabolite. The list of the candidates proposed as being able to stimulate the glycolytic pathway was then examined by, reviewing a dedicated literature.

### Availability of supporting data

The datasets supporting the results of this article are included within the article and its additional files. A supplementary webpage is available at http://KeyRegulatorFinder.genouest.org. As the tool is based on the use of TRANSPATH database, the access to the tool is planned to be on request. The ongoing password is login: logfinder; password: lsfLayLP.

## Abbreviations

PPAR: Peroxisome proliferator-activated receptor; RXR: Retinoid X receptor; TF: Transcription factor; VDR: Vitamin D receptor.

## Competing interests

The authors declare that they have no competing interests.

## Authors’ contributions

PB conceived the methodology, realized the computations, and drafted the manuscript. AS and FG conceived the study and wrote the manuscript. JvM and SL participated in the design of the study and its coordination, and help to draft the manuscript. All authors have read and approved the final manuscript.

## Supplementary Material

Additional file 1: Table S1 Lists of targets used as inputs for analyzing network properties and to evaluate the method accuracy to provide the expected transcription factor and other biologically relevant upstream candidates. Click here for file

Additional file 2: Table S2 Detailed tests using lists of regulated gene targets with a known solution as inputs. Click here for file

Additional file 3: Table S3 Detailed lists of proposed upstream candidates from a list of target genes experimentally proved to be responsive to *PPARA.* Click here for file
