# Supplementary material for: Using a large-scale knowledge database on reactions and regulations to propose key upstream regulators of various sets of molecules participating in cell metabolism
Source: BMC Syst Biol. 2014 Mar 17;8:32. doi: 10.1186/1752-0509-8-32 (PMC4004165; doi:10.1186/1752-0509-8-32)
Supplement: Additional file 1: Table S1 — Lists of targets used as inputs for analyzing network properties and to evaluate the method accuracy to provide the expected transcription factor and other biologically relevant upstream candidates. [file 1752-0509-8-32-S1.docx]

**Suppl. Table 1:** Lists of targets used as inputs for analyzing network properties and to evaluate the method accuracy to provide the expected transcription factor and other biologically relevant upstream candidates

| *PPARA* gene targets^a^ | DE genes in response to PPAR agonists^b^ | Metabolites participating  in glycolysis^c^ |
| --- | --- | --- |
| *Adipor2; Cd36; LEPR; Slc27a1; SLC27A2; SLC27A4; Acot1; Acot7; ACOT12; ACSL1; ACSL3; Acsl4; ACSL5; ACSM3; Acss2; FABP1; Fabp2; FABP3; Fabp4; Fabp5; ACAA2 ; Acadl; ACADM; ACADS ; ACADVL; Acad8 ; Acad9; Acad10; Acot2; Acot9; Acot10; CPT1A; Cpt1b; CPT2; Crat; Dci; Decr1; ETFA; Etfb; ETFDH; HADHA; Hadh; Hadh2; Hibch; SLC25A20; SLC22A5; TXNIP; Ucp2; Ucp3; Acat1; Bdh; FGF21; Hmgcl; HMGCS2; ABCD2; ABCD3; ACAA1A; Acaa1b; Acot3; Acot4; Acot5; Acot8; ACOX1; Crot; Decr2; ECH1; Ehhadh; HACL1; HSD17B4; Peci; Pex11a; ALDH3A1; Aldh3a2; ALDH9A1; Cyp4a1; Cyp4a10; Cyp4a12a ; Cyp4a14; Cyp4f15; Cyp4x1; Acaca; ACACB; AGPAT2; Agpat3; Agpat5; Agpat6; Dgat1; ELOVL5; ELOVL6; Elovl7; FADS1; Fads2; Fasn; GPAM; Hsd17b12; Lpin2; MLYCD; Mogat1; MOD1; Scd1; Scd2; Slc25a10; Srebf1; ADFP; Ces1; Ces3; Cidea; CIDEC; Gos2; Lipa; Lipe; Mgll; Oxpat/Lsdp5; Plin1; PNPLA2; S3-12; ANGPTL4; APOA1; APOA2; APOA5; APOCIII; LIPC; Lipg; Lpl; Lrp4; PCTP; Pltp; Mttp; VLDLR ;ABCA1; ABCB4; Abcb11; Abcg5; Abcg8; Cav1; CYP7A1; Cyp8b1; Cyp27a1 ; FXR ; LXR ; Npc1 ; Rab9 ; Scarb2 ; Slc10a1 ; Slc10a2; AQP3; Aqp7; Aqp9; Fbp2; G6PC; Gpd1; Gpd2; GYK; Gys-2; Ldha; Pcx; PCK1; Pdk1; PDK4; AKR1B10; AKR1C3; CYP1A2; Cyp2a5; CYP2B6; CYP2C8 ; CYP2C9; Cyp2c11; Cyp2c12; Cyp2c29; CYP2J2; CYP3A5; CYP3A7; CYP3A11; Cyp3a43; EPHX2; Gsta3; MGST3; UGT1A9; ABAT; Acmsd; AGXT2; Arg1; ASL; Ass1; CBS; CPS-1; Cth; Got1; Got2; Gls; GLS2; GPT; Hal; Hpd; OAT; ODC1; OTC; PAH; PSAT1; Tat; Apcs; Birc3; Cebpb; Cd68; Crp; Cxcl10/IP10; FGB; Emr1 (F4/80); Icam-1; Ifi47; Igtp; Nfkbia; Il-1; Il-1r1; Il1rn; Il1rap; Il-6; Il-6ra; Il18; Lcn2; Lifr; Ccl2; Ccl3; Mt1; Mt2;Orm2; Orm3; Nfkb1; Pla1a; Saa2; Saa4; Stat1; Stat2; Stat3; Steap4; Tnfα; Traf2; Vcam1* | *ACAA2; ACADL; ACADVL; ACOT2; ACSS2; ADAM12; ALDH9A1; ANK3; APCDD1; ASB4; BTG3; C10orf99; CA6; CACNA1G; CAPN6; CD24; CD36; CDH11; CDK18; CGNL1; CLU; COL18A1; CPT1A; CRABP1; CXCR7; CYP1B1; CYP51A1; DDX3Y; DECR1; DGAT2; DHRS2; DHRS4; DKK3; DPT; ECH1; ECI2; EGLN3; EPHX2; ERCC1; EREG; ETFDH; FABP4; FAH; ;FAM213A; FBP2; FBXW2; FNTB; FZD4; GATA6; GCNT2; GFAP; GK; GLI2; GNG2; GPC4; HADHA; HADHB; HILPDA; HMGCS2; HSD17B11; HSDL2; HTRA3; IGF2; IMPA2; KCNE4; KLF10; LAMA2; LAMA4; LAMB1; LCN2; LGALS9; LIPE; LONP1; LRP5; LSS; MBNL3; ME1; MEG3; METTL7B; MGP; MVD; MYL9; N4BP2L1; NME4; NPNT; NPR3; NXN; ODZ4; OLFM1; PAPSS2; PARM1; PC; PDE1B; PDK1; PDZRN3; PEX11A; PHYH; PKP2; PLIN2; PLIN4; PNPLA2; POPDC3; POSTN; PPARA; PTGS2; PTK7; PTN; PTP4A1; PTPRM; PYCR1; RBMY1A1; RETSAT; RGS4; SCRN2; SERPINA3; SERPINE1; SERPINE2; SEZ6L2; SFRP1; SH3TC1; SLC16A1; SLC22A5; SLC25A20; SLC25A42; SMOC2; SPRR1A; STRA6; SULF2; TGM2; TMEM97; TRIB3; TUBA4A; UBD; UCP2; VAV3* | Glucose  Glucose-6-phosphate  Fructose-6-phosphate  Fructose 1,6-diphosphate  Glyceraldehyde 3 phosphate  Phosphoenolpyruvate  Pyruvate |

^a^ A literature review [20] dedicated to *PPARA* was used, in which 250 gene targets have been indicated as responsive to *PPARA* in different biological processes in the liver. Genes regulated by PPARA in mouse are indicated in lower case, whereas genes regulated in human are capitalized**.**

^b^ A published study [21] of transcriptional profiling of NIH3 cells in the presence of selective peroxisome proliferator-activated receptors (PPAR) agonists indicated 136 differentially expressed (DE) genes as related to *PPARA* effects.

^b^ The metabolites participating in the successive steps in the glycolytic pathway [32] were supposed to have an increased abundance in response to an unknown external factor.
