# Supplementary material for: Using a large-scale knowledge database on reactions and regulations to propose key upstream regulators of various sets of molecules participating in cell metabolism
Source: BMC Syst Biol. 2014 Mar 17;8:32. doi: 10.1186/1752-0509-8-32 (PMC4004165; doi:10.1186/1752-0509-8-32)
Supplement: Additional file 2: Table S2 — Detailed tests using lists of regulated gene targets with a known solution as inputs. [file 1752-0509-8-32-S2.docx]

**Suppl. Table 2.** Detailed tests using lists of regulated gene targets with a known solution as inputs^a^

| known transcription factor | number of  gene targets | mapping in the TRANSPATH database |
| --- | --- | --- |
| db-AR | 9 | G001544 |
| db-ARNT | 4 | G004677 |
| db-ATF1 | 49 | G005792 |
| db-ATF2 | 28 | G000232 |
| db-Atf3 | 1 | G002871 |
| db-ATF3 | 8 | G002871 |
| db-ATF4 | 8 | G002874 |
| db-ATF5 | 1 | G002910 |
| db-ATF6 | 3 | G002673 |
| db-BCL3 | 5 | G004492 |
| db-BCL6 | 8 | G004696 |
| db-BRCA1 | 13 | G002124 |
| db-BRCA2 | 1 | G013814 |
| db-c-Myc | 780 | unknown_molecule |
| db-Cebpa | 10 | G000490 |
| db-CEBPA | 90 | G000490 |
| db-Cebpb | 5 | G002899 |
| db-CEBPB | 45 | G002899 |
| db-Cebpd | 3 | G002533 |
| db-CEBPD | 14 | G002533 |
| db-CEBPE | 6 | G002919 |
| db-Cebpg | 1 | G005930 |
| db-Creb1 | 7 | G004624 |
| db-CREB1 | 116 | G004624 |
| db-CREM | 14 | G000532 |
| db-DP-1 | 1 | unknown_molecule |
| db-E2F | 2 | unknown_molecule |
| db-E2F-1 | 53 | unknown_molecule |
| db-E2F-4 | 118 | unknown_molecule |
| db-Egr1 | 4 | G000505 |
| db-EGR1 | 60 | G000505 |
| db-EGR2 | 4 | G000246 |
| db-EGR3 | 2 | G004597 |
| db-EGR4 | 2 | G004598 |
| db-Elk1 | 1 | G002965 |
| db-ELK1 | 12 | G002965 |
| db-Elk3 | 1 | G003953 |
| db-ELK3 | 1 | G003953 |
| db-ELK4 | 1 | G007337 |
| db-EPAS1 | 3 | G004008 |
| db-ERG | 17 | G004691 |
| db-ESR1 | 49 | G003925 |
| db-ESR2 | 16 | G002763 |
| db-Ets1 | 2 | G000217 |
| db-ETS1 | 87 | G000217 |
| db-Ets2 | 1 | G004761 |
| db-ETS2 | 36 | G004761 |
| db-ETV4 | 27 | G007338 |
| db-Fli1 | 1 | G007339 |
| db-FLI1 | 25 | G007339 |
| db-Fos | 6 | G000218 |
| db-FOS | 31 | G000218 |
| db-FOSB | 1 | G000509 |
| db-GLI | 3 | unknown_molecule |
| db-HIF1A | 40 | G003959 |
| db-HLF | 3 | G003946 |
| db-HOXA10 | 1 | G006209 |
| db-HOXA4 | 1 | G000526 |
| db-HOXA5 | 1 | G000525 |
| db-HOXA7 | 1 | G006215 |
| db-HOXB7 | 3 | G000528 |
| db-HOXC8 | 2 | G001732 |
| db-HOXD3 | 3 | G006236 |
| db-HOXD9 | 1 | G000296 |
| db-Jun | 9 | G000219 |
| db-JUN | 122 | G000219 |
| db-Junb | 1 | G002553 |
| db-JUNB | 7 | G002553 |
| db-Jund | 1 | G000545 |
| db-JUND | 15 | G000545 |
| db-LEF1 | 21 | G013559 |
| db-Myb | 1 | G000220 |
| db-MYB | 15 | G000220 |
| db-MYBL1 | 5 | G006448 |
| db-MYBL2 | 8 | G001215 |
| db-Nfia | 4 | G005248 |
| db-NFIC | 63 | G003997 |
| db-NFIX | 1 | G005247 |
| db-Nfkb1 | 3 | G000356 |
| db-NFKB1 | 108 | G000356 |
| db-NFKB2 | 5 | G006466 |
| db-PAX1 | 4 | G009198 |
| db-PAX2 | 2 | G004686 |
| db-PAX3 | 3 | G003971 |
| db-Pax4 | 1 | G006532 |
| db-PAX5 | 5 | G003921 |
| db-PAX6 | 7 | G001629 |
| db-PAX8 | 4 | G003972 |
| db-PGR | 10 | G002781 |
| db-POU2F1 | 56 | G005327 |
| db-POU2F2 | 17 | G003935 |
| db-POU2F3 | 1 | G009163 |
| db-POU3F1 | 1 | G009112 |
| db-POU3F2 | 2 | G009111 |
| db-POU5F1 | 6 | G002472 |
| db-Ppara | 2 | G002888 |
| db-PPARA | 24 | G002888 |
| db-PPARD | 11 | G001639 |
| db-PPARG | 36 | G002769 |
| db-Rara | 1 | G000591 |
| db-RARA | 32 | G000591 |
| db-RARB | 17 | G000376 |
| db-Rarg | 1 | G000377 |
| db-RARG | 13 | G000377 |
| db-Rel | 1 | G004960 |
| db-REL | 21 | G004960 |
| db-Rela | 2 | G004622 |
| db-RELA | 61 | G004622 |
| db-RELB | 4 | G004623 |
| db-Sfpi1 | 3 | unknown_molecule |
| db-SMAD1 | 8 | G002383 |
| db-SMAD2 | 4 | G002225 |
| db-SMAD3 | 20 | G001918 |
| db-SMAD4 | 19 | G002381 |
| db-SMAD6 | 1 | G002371 |
| db-SMAD7 | 7 | G002306 |
| db-Sp1 | 7 | G004580 |
| db-SP1 | 212 | G004580 |
| db-SP2 | 9 | G009930 |
| db-SP3 | 72 | G009931 |
| db-SP4 | 3 | G003999 |
| db-SPI1 | 58 | G005724 |
| db-Stat1 | 3 | G004625 |
| db-STAT1 | 25 | G004625 |
| db-STAT2 | 1 | G004609 |
| db-Stat3 | 2 | G004610 |
| db-STAT3 | 23 | G004610 |
| db-STAT4 | 8 | G004626 |
| db-Stat5a | 1 | G002606 |
| db-STAT5A | 17 | G002606 |
| db-STAT5B | 15 | G002607 |
| db-STAT6 | 10 | G001089 |
| db-TAL1 | 7 | G001188 |
| db-TFAP2A | 107 | G002615 |
| db-TFAP2B | 2 | G004003 |
| db-TFAP2C | 2 | G004004 |
| db-TP53 | 107 | G000583 |
| db-TP73 | 2 | G005523 |
| db-TP73L | 1 | unknown_molecule |
| db-Usf1 | 4 | G002680 |
| db-USF1 | 89 | G002680 |
| db-Usf2 | 3 | G002993 |
| db-USF2 | 71 | G002993 |
| db-WT1 | 19 | G004120 |
| file-AR | 38 | G001544 |
| file-ARNT | 10 | G004677 |
| file-Atf1 | 39 | G005792 |
| file-ATF1 | 75 | G005792 |
| file-Atf2 | 11 | G000232 |
| file-ATF2 | 33 | G000232 |
| file-Atf4 | 8 | G002874 |
| file-Cebpa | 122 | G000490 |
| file-CEBPA | 212 | G000490 |
| file-Cebpb | 67 | G002899 |
| file-CEBPB | 86 | G002899 |
| file-Cebpd | 8 | G002533 |
| file-CEBPD | 16 | G002533 |
| file-CEBPE | 5 | G002919 |
| file-Cebpg | 5 | G005930 |
| file-Creb1 | 142 | G004624 |
| file-CREB1 | 194 | G004624 |
| file-Crem | 11 | G000532 |
| file-CREM | 19 | G000532 |
| file-Egr1 | 44 | G000505 |
| file-EGR1 | 88 | G000505 |
| file-ELK1 | 26 | G002965 |
| file-EPAS1 | 5 | G004008 |
| file-Esr1 | 39 | G003925 |
| file-ESR1 | 103 | G003925 |
| file-ESR2 | 33 | G002763 |
| file-Ets1 | 64 | G000217 |
| file-ETS1 | 159 | G000217 |
| file-ETS2 | 52 | G004761 |
| file-Etv4 | 37 | G007338 |
| file-ETV4 | 73 | G007338 |
| file-FLI1 | 39 | G007339 |
| file-Fos | 8 | G000218 |
| file-FOS | 41 | G000218 |
| file-Hif1a | 63 | G003959 |
| file-HIF1A | 70 | G003959 |
| file-Hoxa1 | 6 | G001826 |
| file-HOXA1 | 5 | G001826 |
| file-Hoxa11 | 9 | G006210 |
| file-HOXA9 | 13 | G013857 |
| file-Hoxc8 | 34 | G001732 |
| file-Jun | 196 | G000219 |
| file-JUN | 227 | G000219 |
| file-Lef1 | 19 | G013559 |
| file-LEF1 | 30 | G013559 |
| file-Myb | 34 | G000220 |
| file-MYB | 169 | G000220 |
| file-MYBL2 | 9 | G001215 |
| file-Nfia | 36 | G005248 |
| file-Nfic | 76 | G003997 |
| file-NFIC | 122 | G003997 |
| file-Nfkb1 | 120 | G000356 |
| file-NFKB1 | 189 | G000356 |
| file-PAX1 | 5 | G009198 |
| file-Pax2 | 6 | G004686 |
| file-Pax5 | 5 | G003921 |
| file-PAX5 | 13 | G003921 |
| file-Pax6 | 16 | G001629 |
| file-PAX6 | 7 | G001629 |
| file-PAX8 | 6 | G003972 |
| file-PGR | 26 | G002781 |
| file-Pou2f1 | 65 | G005327 |
| file-POU2F1 | 111 | G005327 |
| file-Pou2f2 | 15 | G003935 |
| file-POU2F2 | 24 | G003935 |
| file-Pou5f1 | 11 | G002472 |
| file-POU5F1 | 11 | G002472 |
| file-Ppara | 72 | G002888 |
| file-PPARA | 51 | G002888 |
| file-Ppard | 21 | G001639 |
| file-PPARD | 16 | G001639 |
| file-Pparg | 68 | G002769 |
| file-PPARG | 66 | G002769 |
| file-Rara | 44 | G000591 |
| file-RARA | 93 | G000591 |
| file-Rarb | 30 | G000376 |
| file-RARB | 47 | G000376 |
| file-RARC | 33 | unknown_molecule |
| file-Rarg | 25 | G000377 |
| file-Rel | 5 | G004960 |
| file-REL | 18 | G004960 |
| file-Rela | 41 | G004622 |
| file-RELA | 73 | G004622 |
| file-Sfpi1 | 41 | unknown_molecule |
| file-Smad1 | 10 | G002383 |
| file-SMAD1 | 18 | G002383 |
| file-Smad3 | 20 | G001918 |
| file-SMAD3 | 31 | G001918 |
| file-Smad4 | 8 | G002381 |
| file-SMAD4 | 26 | G002381 |
| file-Sp1 | 319 | G004580 |
| file-SP1 | 352 | G004580 |
| file-SP2 | 6 | G009930 |
| file-Sp3 | 77 | G009931 |
| file-SP3 | 89 | G009931 |
| file-SPI1 | 69 | G005724 |
| file-Stat1 | 11 | G004625 |
| file-STAT1 | 33 | G004625 |
| file-Stat3 | 15 | G004610 |
| file-STAT3 | 32 | G004610 |
| file-Stat5a | 6 | G002606 |
| file-STAT5A | 6 | G002606 |
| file-Stat5b | 11 | G002607 |
| file-STAT5B | 6 | G002607 |
| file-Stat6 | 7 | G001089 |
| file-STAT6 | 11 | G001089 |
| file-Tcfap2a | 196 | unknown_molecule |
| file-TFAP2A | 307 | G002615 |
| file-Tp53 | 28 | G000583 |
| file-TP53 | 267 | G000583 |
| file-TP73 | 5 | G005523 |
| file-Trp53 | 108 | unknown_molecule |
| file-Usf1 | 77 | G002680 |
| file-USF1 | 119 | G002680 |
| file-Usf2 | 53 | G002993 |
| file-USF2 | 88 | G002993 |
| file-Wt1 | 16 | G004120 |
| file-WT1 | 43 | G004120 |
