# Supplementary material for: Using a large-scale knowledge database on reactions and regulations to propose key upstream regulators of various sets of molecules participating in cell metabolism
Source: BMC Syst Biol. 2014 Mar 17;8:32. doi: 10.1186/1752-0509-8-32 (PMC4004165; doi:10.1186/1752-0509-8-32)
Supplement: Additional file 3: Table S3 — Detailed lists of proposed upstream candidates from a list of target genes experimentally proved to be responsive to PPARA. [file 1752-0509-8-32-S3.docx]

**Suppl. Table 3.** Detailed lists of proposed upstream candidates from a list of target genes experimentally proved to be responsive to *PPARA*

1. **Score: coverage**

| **Input:** *PPARA* gene targets^a^ | | | |  | **Input:** DE genes in response to PPAR agonists^b^ | | | |
| --- | --- | --- | --- | --- | --- | --- | --- | --- |
| ID**^b^** | Name | Score | Position |  | ID**^b^** | Name | Score | Position |
| G026063 | MAK(m.s.) | 105 | [1-2] |  | G009452 | TGM2(h) | 44 | [1-29] |
| G026063 | MAK(h) | 105 | [1-2] |  | MO000081916 | **VDR(r):RXR-alpha(r)** | 44 | [1-29] |
| MO000089760 | MAK(h) | 105 | [1-2] |  | MO000040150 | **VDR(h):RXR-alpha(h)** | 44 | [1-29] |
| G019133 | GPD1(h) | 104 | [3-27] |  | G003800 | LRP5(h) | 44 | [1-29] |
| G003512 | TRAF2(h) | 104 | [3-27] |  | MO000046244 | COUP-TF2(r) | 44 | [1-29] |
| MO000093739 | **RXRalpha(m.s.):**  **PPARgamma(x)** | 104 | [3-27] |  | G030899 | MVD(m.s.) | 44 | [1-29] |
| G019174 | HSD17B4(h) | 104 | [3-27] |  | G026063 | MAK(m.s.) | 44 | [1-29] |
| G028451 | PAH(m.s.) | 104 | [3-27] |  | G002868 | Crabp1(m) | 44 | [1-29] |
| MO000093740 | **RXRgamma(m.s.):**  **PPARalpha(x)** | 104 | [3-27] |  | G014428 | Nxn(h) | 44 | [1-29] |
| MO000082320 | SREBP-1a(m.s.) | 104 | [3-27] |  | G026242 | UBD(m.s.) | 44 | [1-29] |
| G030853 | HACL1(m.s.) | 104 | [3-27] |  | G003518 | PTPRM(h) | 44 | [1-29] |
| MO000093228 | **RXRalpha(m.s.):**  **PPARalpha(x)** | 104 | [3-27] |  | G006110 | FZD4(m) | 44 | [1-29] |
| G002349 | SREBF2(h) | 104 | [3-27] |  | MO000019619 | **RXR-alpha(h)** | 44 | [1-29] |
| G027006 | LEPR(m.s.) | 104 | [3-27] |  | G003607 | VAV3(h) | 44 | [1-29] |
| G034915 | ELOVL6(h) | 104 | [3-27] |  | MO000081872 | **VDR(m):**  **RXR-alpha(m)** | 44 | [1-29] |
| MO000046244 | COUP-TF2(r) | 104 | [3-27] |  | MO000021495 | **VDR(h)** | 44 | [1-29] |
| G019838 | PTE1(h) | 104 | [3-27] |  | G026910 | PDE1B(m.s) | 44 | [1-29] |
| MO000115781 | 15-ketosterol | 104 | [3-27] |  | G009383 | SFRP1(h) | 44 | [1-29] |
| G023086 | PCTP(m.s.) | 104 | [3-27] |  | G026675 | Dkk3(m) | 44 | [1-29] |
| G027613 | NPC1(ha) | 104 | [3-27] |  | G009993 | NR2F2(r) | 44 | [1-29] |
| G004609 | STAT2(h) | 104 | [3-27] |  | G005127 | TUBA1(h) | 44 | [1-29] |
| G019300 | OAT(h) | 104 | [3-27] |  | G006129 | GLI2(h) | 44 | [1-29] |
| G009993 | NR2F2(r) | 104 | [3-27] |  | G019156 | HADHA(h) | 44 | [1-29] |
| G023262 | FADS1 | 104 | [3-27] |  | MO000117410 | TR2-11 isoform1(m) | 44 | [1-29] |
| MO000093229 | **RXRalpha(m.s.):**  **PPARdelta(x)** | 104 | [3-27] |  | G027388 | ADAM12(m.s.) | 44 | [1-29] |
| G034745 | ELOVL5(h) | 104 | [3-27] |  | G003064 | CD24(h) | 44 | [1-29] |
| MO000093742 | **RXRgamma(m.s.):**  **PPARgamma(x)** | 104 | [3-27] |  | MO000089760 | MAK(h) | 44 | [1-29] |
| MO000093741 | **RXRgamma(m.s.):**  **PPARdelta(x)** | 104 | [3-27] |  | G009905 | NR2C1(h) | 44 | [1-29] |
|  |  |  |  |  | G006666 | RGS4(m) | 44 | [1-29] |

1. **Score: specificity**

| **Input:** *PPARA* gene targets^a^ | | | |  | **Input:** DE genes in response to PPAR agonists^b^ | | | |
| --- | --- | --- | --- | --- | --- | --- | --- | --- |
| ID**^b^** | Name | Score | Position |  | ID**^b^** | Name | Score | Position |
| MO000054360 | **PXRisoform1A(h):**  **RXRalpha(h)** | 2.999 | 1 |  | MO000094760 | TGFbeta(h) | 1.997 | 1 |
| MO000095043 | **VDR(h):**  **RXRalpha(m.s.)** | 1.999 | 2 |  | MO000058219 | activin(m.s.) | 1.997 | 2 |
| MO000080098 | P**PARdelta(m):**  **RXR-alpha(h)** | 1.998 | 3 |  | MO000033840 | **PPARalpha(h):**  **RXRalpha(h)** | 1.995 | 3 |
| MO000118460 | 5alpha-cyprinol | 1.993 | [4-5] |  | MO000093228 | **RXRalpha(ms):**  **PPARalpha(x)** | 1.976 | [4-7] |
| MO000118461 | 5alpha-bufol | 1.993 | [4-5] |  | MO000093741 | **RXRgamma(ms)**  **PPARdelta(x)** | 1.976 | [4-7] |
| MO000114980 | ursodeoxycholate | 1.992 | 6 |  | MO000093740 | **RXRgamma(ms)**  **PPARalpha(x)** | 1.976 | [4-7] |
| MO000089190 | SREBP-1c(m.s.) | 1.988 | 7 |  | MO000093229 | **RXRalpha(m.s.):PPARdelta(x)** | 1.976 | [4-7] |
| MO000109503 | geranylgeraniol | 1.986 | 8 |  | MO000093739 | **RXRalpha(m.s.):PPARgamma(x)** | 1.974 | [8-10] |
| MO000033847 | **PPARgamma(r):RXR-alpha(r)** | 1.982 | [9-10] |  | MO000093742 | **RXRgamma(ms)**  **PPARgamma(x)** | 1.974 | [8-10] |
| G002870 | Esrra(m) | 1.979 | 11 |  | MO000033685 | **PPARgamma2 (m)** | 1.974 | [8-10] |
| MO000046946 | **RXRgamma(m.s.)** | 1.978 | [12-14] |  | G029216 | LAMA2 | 0.999 | [11-14] |
| MO000082389 | HIC-5(m) | 1.978 | [12-14] |  | G024531 | COL18A1 | 0.999 | [11-14] |
| MO000114983 | deoxycholate | 1.978 | [12-14] |  | G028813 | LAMA4 | 0.999 | [11-14] |
| MO000083304 | SREBP-2(h) | 1.976 | 15 |  | G028217 | NPR3 | 0.999 | [11-14] |
| MO000048171 | insulin(m.s.) | 1.971 | 16 |  | G018632 | SERPINA3(h) | 0.993 | [15-20] |
| MO000046512 | CAR(m.s.) | 1.964 | 17 |  | G028764 | CDH11 | 0.993 | [15-20] |
| MO000024696 | CBF(2)(r) | 1.961 | 18 |  | G030577 | DPT(m.s.) | 0.993 | [15-20] |
| MO000114979 | lithocholate | 1.943 | [19-21] |  | G002527 | SPRR1A(h) | 0.993 | [15-20] |
| MO000081023 | Alien(h) | 1.943 | [19-21] |  | G029465 | PRSS15(h) | 0.993 | [15-20] |
| MO000121739 | T3R-alpha(c):Alien(h) | 1.943 | [19-21] |  | G027270 | APCDD1(m.s.) | 0.993 | [15-20] |
| MO000025607 | T3R-alpha(c) | 1.943 | [19-21] |  | G019390 | PTK7(h) | 0.998 | [21-29] |
| MO000026692 | GATA-4(r) | 1.926 | 22 |  | G023189 | LCN2(m.s.) | 0.998 | [21-29] |
| MO000026496 | foxm1-isoform2(h) | 1.896 | [23-24] |  | MO000109018 | (JunD(h))2 | 0.998 | [21-29] |
| MO000026497 | foxm1-isoform4(h) | 1.896 | [23-24] |  | MO000035753 | JunB(h):JunB(h) | 0.998 | [21-29] |
| G004005 | FOXM1(h) | 1.887 | 25 |  | G028337 | CMKOR1(m.s.) | 0.998 | [21-29] |
| G025842 | ABCD2 | 0.999 | [26-29] |  | G032274 | PDK1(h) | 0.998 | [21-29] |
| MO000154173 | Sox-8(m.s.) | 0.999 | [26-29] |  | G020617 | ACSS2(h) | 0.998 | [21-29] |
| G006947 | CYP2C9(h) | 0.999 | [26-29] |  | G022399 | DECR1(m.s.) | 0.998 | [21-29] |
| G020752 | ABCG8(h) | 0.999 | [26-29] |  | G025087 | RBMY1A1(h) | 0.998 | [21-29] |
| G000382 | SAA2(h) | 0.998 | [30-46] |  | G041914 | PYCR1(h) | 0.998 | [21-29] |
| G004731 | SLC10A2(h) | 0.998 | [30-46] |  | G027454 | STRA6(m.s.) | 0.998 | [21-29] |
| G019470 | SLC10A1(h) | 0.998 | [30-46] |  | G046706 | SERPINE2(h) | 0.998 | [21-29] |
| G031601 | LRP4(h) | 0.998 | [30-46] |  | MO000007830 | JunB(h) | 0.998 | [21-29] |
| G023189 | LCN2(m.s.) | 0.998 | [30-46] |  | G019108 | FNTB(h) | 0.998 | [21-29] |
| G010669 | CD68 | 0.998 | [30-46] |  | G004776 | HMGCS2(h) | 0.998 | [21-29] |
| G028911 | STEAP4(m.s.) | 0.998 | [30-46] |  | G026526 | MBNL3(m.s.) | 0.998 | [21-29] |
| G024569 | AQP9(m.s.) | 0.998 | [30-46] |  |  |  |  |  |
| G020655 | AKR1B10(h) | 0.998 | [30-46] |  |  |  |  |  |
| G047538 | CES3(h) | 0.998 | [30-46] |  |  |  |  |  |
| G018937 | APCS(h) | 0.998 | [30-46] |  |  |  |  |  |
| G024499 | ACAD8(m.s.) | 0.998 | [30-46] |  |  |  |  |  |
| G030315 | GYS2(m.s.) | 0.998 | [30-46] |  |  |  |  |  |
| G021667 | EMR1(m) | 0.998 | [30-46] |  |  |  |  |  |
| G030184 | AQP3(m.s.) | 0.998 | [30-46] |  |  |  |  |  |
| G004760 | Slc27a1(m.s.) | 0.998 | [30-46] |  |  |  |  |  |
| G028004 | ACOT9(m.s.) | 0.998 | [30-46] |  |  |  |  |  |

^a^ The lists consisted of targets of *PPARA* reviewed in a dedicated literature on human and mouse (250 regulated genes; [20]) or experimentally identified as responsive to peroxisome proliferator-activated receptors (PPAR) agonists in cell culture (136 transcripts; [21]).

^b^ Candidates were proposed and ranked according to their ability to explain the greatest number of targets (coverage) or a tradeoff between the number of regulated targets and the total number of regulated molecules (specificity). Candidates of similar score were indicated with the corresponding range of ranks in brackets. TRANSPATH IDs [13] begin with “MO” for metabolites and proteins and “G” for genes. m.s.: mouse; h: human; r: rat. This table shows that many biologically relevant candidates in PPAR pathways (bold face) were heterodimer protein complexes, which underlines the necessity to merge metabolic and genetic information to understand the whole cell regulation.
